# Object Localization Does Not Imply Awareness of Object Category at the Break of Continuous Flash Suppression

**DOI:** 10.3389/fnhum.2017.00312

**Published:** 2017-06-15

**Authors:** Florian Kobylka, Malte Persike, Günter Meinhardt

**Affiliations:** Research Methods and Statistics, Department of Psychology, Institute of Psychology, Johannes Gutenberg University MainzMainz, Germany

**Keywords:** binocular rivalry, continuous flash suppression, visual awareness, face inversion effect, object recognition

## Abstract

In continuous flash suppression (CFS), a dynamic noise masker, presented to one eye, suppresses conscious perception of a test stimulus, presented to the other eye, until the suppressed stimulus comes to awareness after few seconds. But what do we see breaking the dominance of the masker in the transition period? We addressed this question with a dual-task in which observers indicated (i) whether the test object was left or right of the fixation mark (localization) and (ii) whether it was a face or a house (categorization). As done recently Stein et al. (2011a), we used two experimental varieties to rule out confounds with decisional strategy. In the terminated mode, stimulus and masker were presented for distinct durations, and the observers were asked to give both judgments at the end of the trial. In the self-paced mode, presentation lasted until the observers responded. In the self-paced mode, b-CFS durations for object categorization were about half a second longer than for object localization. In the terminated mode, correct categorization rates were consistently lower than correct detection rates, measured at five duration intervals ranging up to 2 s. In both experiments we observed an upright face advantage compared to inverted faces and houses, as concurrently reported in b-CFS studies. Our findings reveal that more time is necessary to enable observers judging the nature of the object, compared to judging that there is “something other” than the noise which can be localized, but not recognized. This suggests gradual transitions in the first break of CFS. Further, the results imply that suppression is such that no cues to object identity are conveyed in potential “leaks” of CFS (Gelbard-Sagiv et al., 2016).

## 1. Introduction

Binocular rivalry is an intriguing phenomenon which stimulated several lines of research on unconscious processing. In binocular rivalry, two dissimilar images, presented to corresponding regions of both eyes, are not perceptually fused, but get access to visual awareness in temporal alternation. The alternations in conscious perception are thought to reflect competition among the neural structures involved in processing the two stimulus alternatives (Blake and Logothetis, 2002). In traditional studies of binocular rivalry, competing stimuli with comparable low-level attributes are used, which leads to comparable durations of dominance epochs (Blake, 1989).

Some years ago Tsuchiya and Koch (2005) introduced Continuous Flash Suppression (CFS), a potent technique to preclude a static image from getting access to visual awareness. In this technique a high contrast, colored noise masker, flickering with a temporal frequency of about 10 Hz, is presented to one eye, while the other eye is stimulated with a static image. The dynamic masker occupies visual awareness right from the start of the trial, and its initial dominance epoch lasts up to more than 10 times longer compared to traditional rivalry. Moreover, even large maskers do not suffer from piecemeal rivalry (Blake et al., 1992). The seemingly complete suppression of the test stimulus by the dynamic masker right from trial start made the CFS technique attractive for studying visual processing in the absence of awareness. Using CFS it was shown that the suppressed image, albeit not being consciously perceived, exerts visual aftereffects in stimulus attributes like orientation (Kanai et al., 2006) and contrast (Shin et al., 2009), and also visual priming effects (Almeida et al., 2008). In the latter study object primes suppressed by the CFS masker speeded responses to probe objects of the same specific object category as the prime in the test phase after CFS. This suggested priming effects at the level of object category. However, later studies revisiting the question of object category specific priming in CFS found that primes with some shape similarity to the tested object category also elicited priming effects (Sakuraba et al., 2012), while tests of object category specific priming effects failed (Hesselmann et al., 2016). Hence, alternative explanations in terms of lower level shape similarity could not be ruled out.

The conclusions about unconscious processing under CFS conditions in adaptation and priming techniques critically hinge on the assumption that the test stimulus is completely suppressed by the masker (Yang et al., 2014). That is, it must be ascertained that suppression is not “leaky,” allowing single stimulus attributes to escape from suppression. To ascertain complete suppression is challenging, and, indeed, the literature on partial awareness in CFS is growing (Carlson and He, 2000; Hong and Blake, 2009; Zadbood et al., 2011; Gelbard-Sagiv et al., 2016). In contrast to adaptation and priming, the “breaking continuous flash suppression” (b-CFS) technique measures the time it takes for a test stimulus to have its first access to awareness. In this paradigm, the test stimulus is displayed to one eye, and left or right from the fixation mark, while the CFS masker is presented to the other eye. The observer responds when she or he notices the emerging stimulus by indicating whether it begins to appear to the left or to the right. Using this technique, Jiang et al. (2007) showed that upright faces break suppression earlier than inverted faces. These results suggested unconscious processing at the level of face representations in the suppression period, since, in conscious processing, the inversion effect has been shown to be a marker of face-tuned processing in a large variety of tasks (Yin, 1969; Thompson, 1980; Diamond and Carey, 1986; Tanaka and Sengco, 1997). The finding of shorter suppression durations for upright compared to inverted faces is meanwhile a well established finding with the b-CFS technique, and has been replicated several times (Yang et al., 2007; Zhou et al., 2010; Stein et al., 2011a, 2012). Studying other object categories, like birds, dogs and trees, did not show substantial inversion effects under CFS conditions (Stein et al., 2012). Particularly, houses did not show inversion effects (Zhou et al., 2010). Strong inversion effects for faces in contrast to modest ones for novel non-face objects fairly well agree with the results for conscious category-specific object processing (Yin, 1969; Yovel and Kanwisher, 2004, 2008).

It is tempting to consider the duration of the suppression epoch as an index for unconscious processing, since this duration varies with higher and lower level stimulus attributes (see recent comprehensive reviews of Yang et al. (2014) and Gayet et al. (2014). However, the different lengths of the suppression period may also arise as a result of processing differences during the transition period, in which the test stimulus gradually gains access to awareness. Since the stimulus has already started to get access to awareness, these processes concern conscious prossing, similar to detection of signals in noise, but hardly unconscious processing (see discussion of this point in Gayet et al., 2014). Recent findings showing that relatively crude visual shape processing is sufficient to obtain inversion effects in b-CFS durations support this conjecture (Stein et al., 2011b). Therefore, the b-CFS paradigm cannot provide unequivocal evidence for unconscious processing (Stein et al., 2011a).

But how may the process of getting access to awareness be conceived? In view of the evidence for unconscious processing at the level of object category one might surmise that perception of the noise masker is replaced by a conscious perception of a meaningful object in the moment the observer indicates its presence correctly. Hence, knowing “where” might imply knowing “what.” Mudrik et al. (2013) studied the accuracy of both localization and within-class object categorization. They found that categorization of faces as famous or non-famous was consistently worse than correctly localizing them^1^. Critically, the authors found that brief periods of partial awareness occurred during CFS that allowed to localize the test stimulus, but these brief “leaks” of CFS did not allow identification of crucial object related attributes. Albeit a more advanced level of object representation is necessary for discriminating famous and non-famous faces, compared to merely judging the basic level category, these results might indicate that object-related attributes are not perceptually available when the observer correctly localizes the test stimulus. The finding might also indicate that the transition into conscious perception in CFS begins with a raw segmentation which continuously refines, thus enabling object categorization and, later, identification. This resembles assumed processing steps in object recognition, whereby segmentation at different levels precedes object categorization (Marr, 1982). The temporal order of processing steps in object recognition, however, is a matter of controversial debate (Grill-Spector and Kanwisher, 2005; Mack et al., 2008).

A first step into investigating which kind of information is accessible to the observer at the break of CFS is to study whether localization goes along with basic level object categorization, since basic level categorization can be regarded as the entry level of object related processing, while within-class categorization and discrimination at the individual level require more elaborated processing steps (Grill-Spector and Kanwisher, 2005). That is, we need to clarify whether observers can distinguish a face from a house, or a dog from a car, etc., at the moment when she or he indicates the location of the emerging stimulus correctly. Methodologically, it is challenging to measure localization along with object categorization, since the observer might resort to different decisional strategies for either judgment. For example, one may run b-CFS trials with two different instructions, one requiring to indicate the objects' location and the other its category. If it turns out that the b-CFS durations are longer for categorization compared to localization one cannot unambiguously conclude that localization is feasible before categorization, because the observers might use different response criteria for either judgment. In particular, coincident localization and categorization, but use of different response criteria would be indicated by a duration-accuracy trade-off, whereby shorter b-CFS durations for localization go along with less accuracy rates than in categorization. On the other hand, shorter b-CFS durations which go along with higher or same accuracy rates than in categorization would indicate that object localization can be done earlier than basic level object categorization.

Another technique that avoids problems arising from subjective criterion settings was used recently (Stein et al., 2011a). Using upright and inverted faces as test stimuli, the authors (see Stein et al., 2011a, Experiment III) stopped presentation of test face and CFS masker at several definite durations within the first 2 s and asked the observers to judge test stimulus location as good as possible. Plotting accuracy as a function of CFS duration showed monotonously increasing accuracy rates, which reflected a clear face inversion effect. It is intriguing to employ this technique combined with a dual response task. When the display is terminated the observers are asked to respond to both aspects, the “where” and the “what” of the test stimulus. If the observers are less accurate in categorization compared to localization, then this would indicate that low level stimulus attributes, which reveal object presence in noise, are released from suppression earlier than higher level cues to its nature.

In the present study we used both techniques, the self-paced termination of the CFS epoch in b-CFS, and the terminated CFS technique of Stein et al. (2011a) to address the question whether object localization precedes basic level object categorization under CFS conditions. In particular, we asked whether both experimental techniques converge to a unique picture of what a subject sees in the moment she or he reports that CFS is broken.

## 2. The present study

The present study comprises two experiments with identical experimental setup. Faces and houses, presented upright and inverted, were used as test stimuli. In Experiment I the classical b-CFS technique was used to measure b-CFS durations for test stimulus localization (task I) and categorization (task II) in the usual self-paced manner. In Experiment II five CFS durations were selected within the interval up to 2 s, and presentation was terminated by the computer. After the presentation interval the subjects consecutively indicated stimulus location relative to fixation and then its object category, or vice versa. The same subjects participated in both experiments, which were run in one experimental session. Half of the subjects started the experimental session with Experiment I, the other half with Experiment II.

## 3. Experiment I: self-paced CFS

### 3.1. Methods and materials

#### 3.1.1. Apparatus

The experiments were executed with standard desktop computers using Mathlab 2014b runtime units. Subjects viewed stimuli through a custom built mirror stereoscope from a distance of approximately 60 cm. Responses were given via external Cedrus RB-830 response pads with internal timers for response time measurements. Patterns were displayed on NEC MultiSync E222W TFT displays at 1,920 × 1,080 pixel resolution and a refreshing rate of 60 Hz. No gamma correction was used. Sennheiser HD 201 headphones were used for acoustical feedback. The entire experimental session was conducted in a standard laboratory without windows, constant temperature, dimmed light and a maximum of three persons in the room (two participants and the examiner). Stimuli were prepared in Adobe Photoshop CS5. Data were processed in Microsoft Excel 2013 and analyzed with Statistica 12.

#### 3.1.2. Stimuli

**Figure 1:**
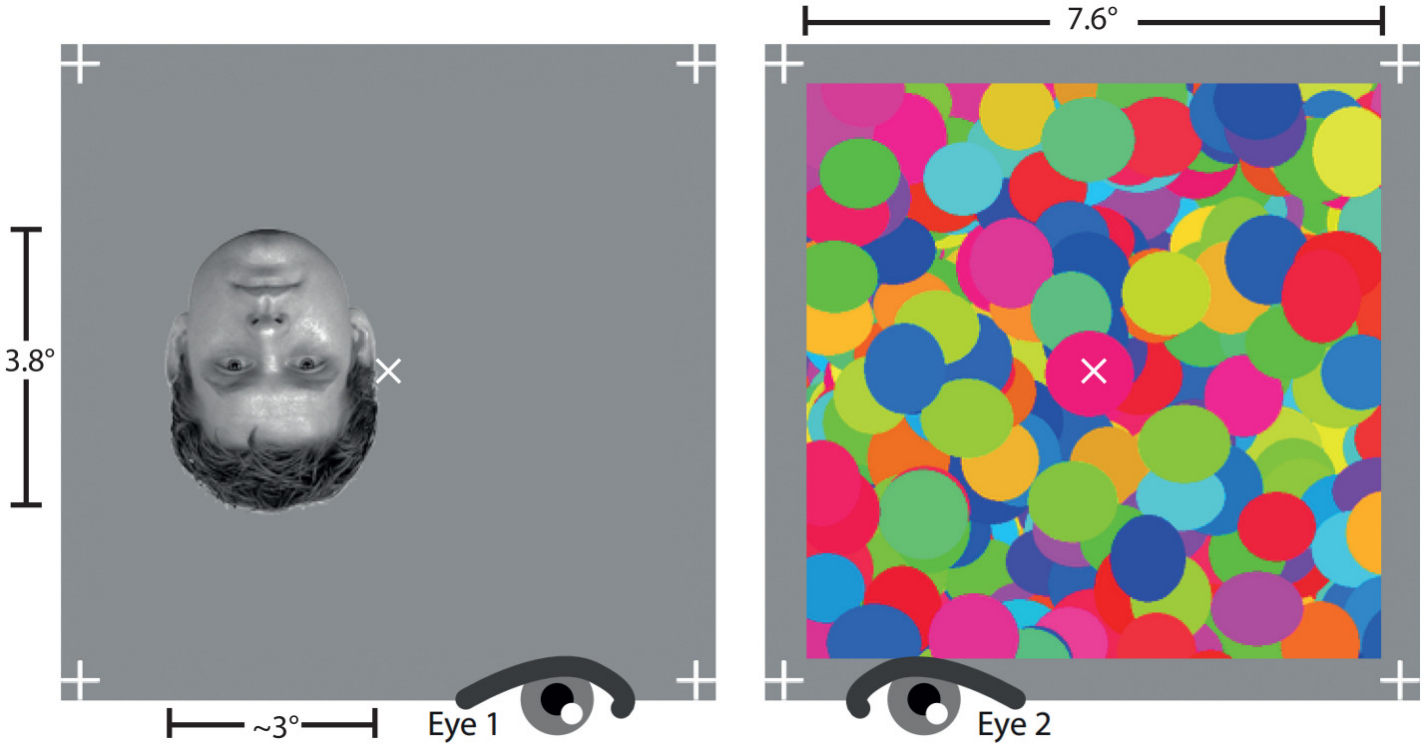
Example of a CFS stimulus arrangement with the test stimulus (here: inverted face) presented to the left eye and the dynamic noise masker presented to the right eye.

Faces and houses were used as test stimuli. Eight face images were selected from the Radboud Face Database (Langner et al., 2010). Eight house images were sampled from different internet sources. Image selection and editing was identical to the procedures used by Persike et al. (2014). We used four male and four female faces with neutral emotional expressions. House images all contained a door, windows, and either a gable structure or a part of the pitched roof. The images were manipulated by converting them into grayscale, removing picture background, scaling them to a height of 150 pixels (3.8° visual angle) and a width of 108–132 pixels (2.73°–3.34° visual angle) depending on the proportions of each face or house. Images were rotated by 180° to create their inverted counterparts. Luminance histograms of all images were equalized with a simple quantile transformation. Root mean square contrast for all images was 0.176 in normalized units. Each image was presented in an area sized 267 × 267 pixels (6.7° × 6.7° visual angle). The area was marked by four crosses at the edges of the frame and another cross in its center to help observers maintain eye vergence in the dichoptic stimulus arrangement. The central cross also served as a fixation marker during trials. Images were presented either right or left of the fixation marker, with an offset of 50 pixels. The CFS mask consisted of variations of a picture with overlapping colored circles (see Figure 1), changing randomly at a constant rate of 10 Hz. The size of the mask was 300 × 300 pixels. Stimulus arrangements were displayed on a gray screen canvas with a luminance of 93.2 cd/m^2^, matching the mean image luminance. Additionally, a black-white random dot pattern with a grain resolution of three pixels, having the same size as the CFS masker, was presented to both eyes after stimulus and mask presentation to avoid afterimages. Figure 1 illustrates an example of the test stimulus-mask arrangement.

#### 3.1.3. Subjects

Twenty-five volunteers (15 female, age range 18–35 years, mean age 24.9 years, SD 4.4 years) participated in the experiments. Most of them were undergraduate students of psychology (17), one was a pupil, and one was one of the authors (FK). The remaining participants were students in other majors. The participants received either payment (8 euros) or course credit, except the author FK. All participants had normal or corrected-to-normal vision. The study adhered to the Declaration of Helsinki. In particular, written consent was given that participation was voluntarily, that data were collected and saved anonymously, and that there were no negative consequences if participants wanted to abandon participation at any given point. The experimental procedures were approved by the local ethics committee at the Johannes Gutenberg University Mainz. Previously to the experiment participants were informed about the procedures and general intention of the study and gave written consent for participation. After the measurements were complete a summary and an explanation of their data was provided to each participant.

#### 3.1.4. Procedure

Half of the subjects started with the localization task, the other half with the categorization task, chosen at random for each subject individually by the experimenter. The two different tasks were run in separate blocks, separated by a 5 min break. The participants were briefed about dichoptic viewing conditions, and adjusted the seat and the stereoscope according to their height, ocular anatomy and vergence disposition, so that they were able to maintain a comfortable position. Prior to each experimental block the stereoscope was calibrated anew to ensure congruency of stimulus and mask displays. Trial by trial acoustical feedback of correctness was given via headphones to give better opportunity to control response bias (Meinhardt et al., 2014). A brief “tack” tone was used for correct and a “tacktack” tone for incorrect responses. In the localization task the participants were instructed to respond by button press when a stimulus was seen left or right of the central fixation cross. For the categorization task they were instructed to indicate whether they saw a face or a house. The participants were alerted to responding instantly when they were reasonably certain that a stimulus appeared. The participants were administered 16 practice trials with new face and house test stimuli which were not used in the experiment. If more than 2 errors occurred, subjects practiced again with a block of 8 trials, until no error occurred. In both experiments an experimental trial started with the CFS masker presented to one eye, chosen at random, and an empty box with fixation mark presented to the other (see Figure 1). After 650 ms plus a random onset delay ranging up to 1,800 ms the test stimulus started to fade-in linearly for a time interval of 1 s. The clock for measuring b-CFS duration was started after the first refresh cycle, which displayed the test stimulus with a contrast of 1/60. Presentation of test stimulus and CFS masker was terminated when the subject pressed a response button, or after 20 s if no response was given up to this time limit. Either event started presentation of black and white random dot patterns to the left and right eye display fields. The four conditions (2 stimulus categories × 2 orientations) were measured with 24 replications, which means that each subject judged 96 trials for each task.

#### 3.1.5. Data analysis and outlier clearing

Accuracy rates and the durations of correct responses were analyzed. We applied outlier control to the b-CFS durations collected for each individual subject by using Tukey's method (Tukey, 1977), which resorts to the inter-quartile range (IQR) to identify outliers. According to this method, values smaller than the 1st quartile − 1.5 × IQR and larger than the 3rd quartile + 1.5 × IQR are removed. Outlier removal with this procedure mostly concerned durations above 10 s (see also Stein et al. (2011a)), and no times below the first quartile. Analyzing the number of durations *n* that remained in the duration samples of the subjects had a median of *q*_0.5_ = 21, a first quartile of *q*_0.25_ = 20 and a third quartile of *q*_0.75_ = 22. This means that, after removing durations for wrong responses and clearing the remaining values of outliers, still 20–22 values of the 24 measurements remained in the individual samples, which was considered as a solid basis for calculating a mean duration for each subject in each experimental condition, which is referred to as the raw duration measure, *D*, in the following. However, besides positive outliers (extraordinarily long b-CFS durations), another issue is positive skewness, since epoch durations in binocular rivalry are known to be positively skewed (Logothetis et al., 1996). Because sample sizes of 20–22 durations may not be enough to establish normality of the sample means according to the central limit theorem, we calculated log-transformed durations, since the log-transform is an efficient means to reduce positive skewness (Ratcliff, 1993; Gayet and Stein, 2017). The raw duration measure *D*, the log_10_ transforms of *D*, and the accuracy data were analyzed for its distribution properties and entered statistical testing with a 2 (Task; localization or categorization) × 2 (Stimulus; face or house) × 2 (Orientation; upright or inverted) repeated measurements ANOVA.

### 3.2. Results

#### 3.2.1. Results for b-CFS durations

First, the within-cell data were checked for skewness and normality. Normality was assessed with with Shapiro-Wilk W test (Shapiro and Wilk, 1965), which is regarded as sensitive even to moderate violations of normality and is applicable even for smaller samples (see Johnson and Wichern, 2002, p. 182). The results are shown in Table 1. For the raw duration measure *D* all the within-cell distributions were positively skewed. Violation of normality occurred in 5 from 8 cells, while stronger violations were associated with larger positive skewness. For the log_10_(*D*) measure noticeable positive skewness was observed only for one cell, and there were no violations of normality. While these results show that the log-transformed duration data are better suited for parametric testing, we report testing results for both measures.

**Table 1:** Results of testing the cell distributions of Experiment 1 for normality for the raw durations measure, *D*, and for the log-transformed durations, log_10_(*D*).

**Task**	**Localization**				**Categorization**			
**Stimulus**	**Face**		**House**		**Face**		**House**	
**Orientation**	**Upright**	**Inverted**	**Upright**	**Inverted**	**Upright**	**Inverted**	**Upright**	**Inverted**
**RAW DURATION MEASURE (*****D*****)**								
Skewness	0.734	0.920	1.257	0.759	1.046	1.448	2.368	0.859
Shapiro-Wilk *W*	0.912	0.925	0.886	0.912	0.924	0.886	0.733	0.923
*p*	0.033	0.067	0.009	0.034	0.063	0.009	0.000	0.059
Violation of normality	^*^	−	^*^	^*^	−	^*^	^*^	−
**log_10_(*D*) DURATION MEASURE**								
Skewness	−0.012	−0.073	0.197	−0.050	0.248	0.199	0.933	0.017
Shapiro-Wilk *W*	0.948	0.967	0.972	0.948	0.978	0.982	0.928	0.975
*p*	0.229	0.586	0.698	0.222	0.849	0.927	0.079	0.776
Violation of normality	−	−	−	−	−	−	−	−

*The table shows skewness, the Shapiro-Wilk W statistic, and probability of the observed deviation given the null hypothesis (normality), p. Violation of normality on any level of significance equal or below α = 0.05 is indicated by an asterisk*.

**Figure 2:**
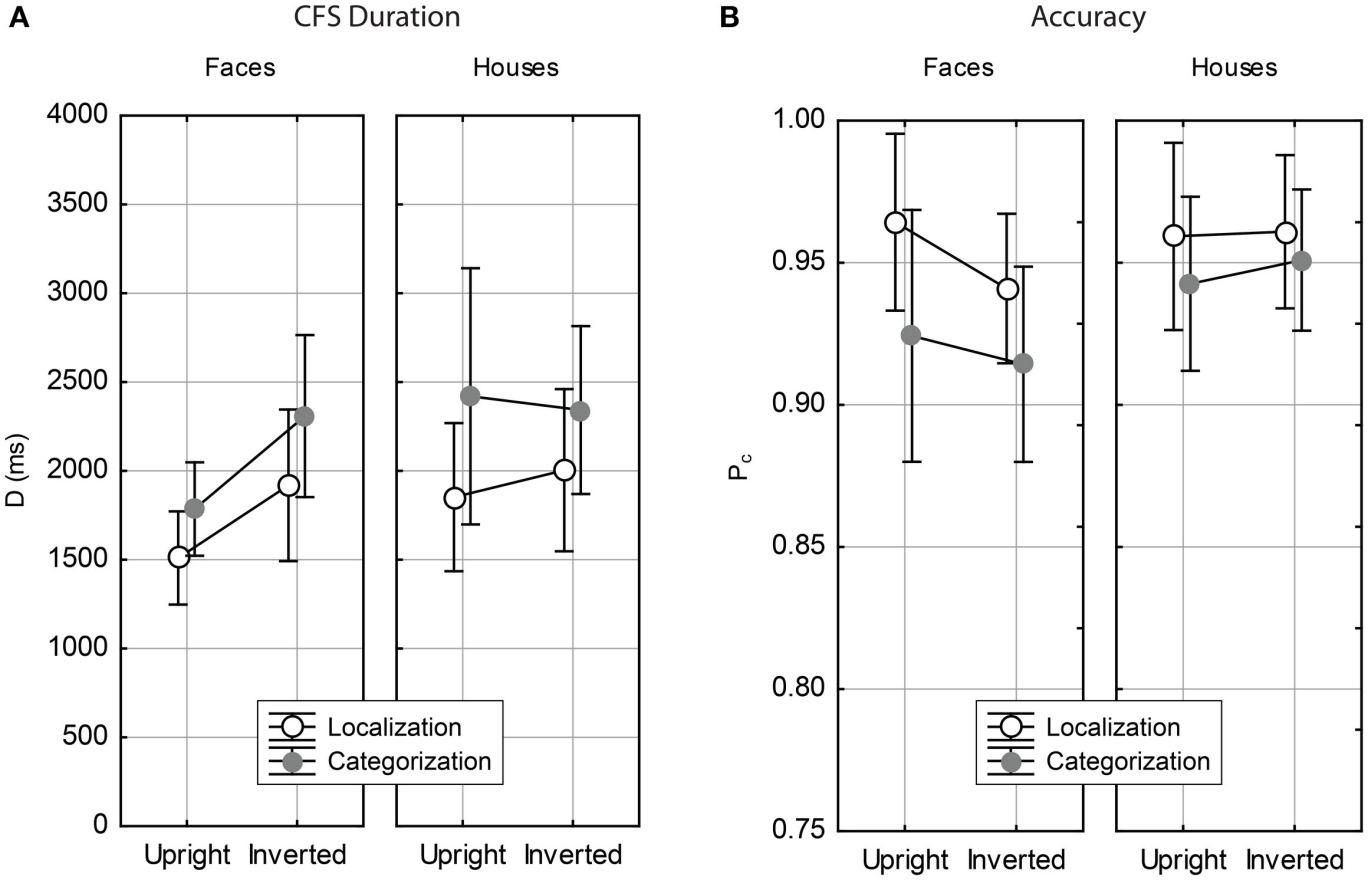
Results for Experiment I (self-paced CFS). Mean b-CFS durations are shown in **(A)**, and mean accuracy rates are shown in **(B)**, arranged as as Task × Orientation interaction plots. Error bars indicate 95% confidence intervals of the means.

Analyzing the raw duration data *D* with ANOVA showed main effects in all three factors Task, Stimulus and Orientation. The task effect [Δ*D* = 393 ms, *F*_(1, 24)_ = 6.34, *p* < 0.02] indicated that correct stimulus categorization needed more time to break CFS than correct stimulus localization. Further, faces broke CFS earlier than houses [Δ*D* = 274 ms, *F*_(1, 24)_ = 6.76, *p* < 0.02], as did upright stimuli compared to inverted [Δ*D* = 252 ms, *F*_(1, 24)_ = 14.42, *p* < 0.001]. Further, the Stimulus × Orientation interaction was significant [*F*_(1, 24)_ = 5.55, *p* < 0.03]. No further interactions reached significance. In Figure 2A the duration data are illustrated as Task × Orientation interaction plots, one for each stimulus category. The plot illustrates that the Stimulus × Orientation interaction indicates an inversion effect for faces, but not for houses. This results was independent of the task (absence of the Stimulus × Orientation × Task interaction). The differential inversion effects with respect to stimulus category were confirmed by pairwise comparisons. Inverted faces took longer to break CFS than upright faces [Δ*D* = 466 ms, *F*_(1, 24)_ = 20.76, *p* < 0.001], while there was no orientation related effect on CFS duration for houses [Δ*D* = 37 ms, *F*_(1, 24)_ = 0.09, *p* = 0.762]. Testing across stimulus category showed that upright faces broke CFS much faster than upright houses [Δ*D* = 488 ms, *F*_(1, 24)_ = 7.86, *p* < 0.01], while there was no difference in CFS duration for both stimulus categories in inverted presentation [Δ*D* = 60 ms, *F*_(1, 24)_ = 0.42, *p* = 0.523].

Analyzing the log-transformed duration data with ANOVA showed the same results than the ANOVA for the raw *D* measure, while the effects were slightly more pronounced. Table 2 shows the results of the main effects and the stimulus specific tests of the inversion effect for both measures. Comparing Cohen's *d* effect size measure showed moderately larger effect sizes for the log_10_(*D*) measure. The face inversion effect, however, reached the same effect size of about *d* = 0.9 in both measures.

**Table 2:** Results of paired tests for the main effects of task, stimulus and orientation, as well as orientation effects for faces and houses, for the raw duration measure, *D*, and log-transformed durations, log_10_(*D*).

	**Raw duration measure (*****D*****)**					**log**_**10**_(***D***) **duration measure**				
	**Δ**	***t***	***df***	***p***	***d***	**Δ**	***t***	***df***	***p***	***d***
Task	393	2.52	24	0.019	0.50	0.092	3.29	24	0.003	0.66
Stimulus	274	2.60	24	0.016	0.52	0.041	2.59	24	0.016	0.52
Orientation	252	3.80	24	<0.001	0.76	0.053	4.54	24	<0.001	0.91
Orientation (Faces)	466	4.56	24	<0.001	0.91	0.087	4.59	24	<0.001	0.92
Orientation (Houses)	37	0.31	24	0.762	0.06	0.018	1.29	24	0.210	0.26

*The table shows the difference in the measure, Δ, t-statistic, degrees of freedom, p-value, and Cohen's d*.

#### 3.2.2. Accuracy

The proportion correct rates obtained in Experiment I are shown in Figure 2B. The data show that accuracy was generally high. Note that, with *N* = 24 trial replications, one error corresponds to *P*_*c*_ = 0.958 and two errors to *P*_*c*_ = 0.917. Hence, the proportion correct rates reflect that about one error was made in localization on the average, while about one to two errors occurred in categorization. ANOVA of the proportion correct measure revealed main effects of Task and Stimulus, but none of Orientation. The task effect [Δ*P*_*c*_ = 0.023, *F*_(1, 24)_ = 11.38, *p* < 0.01] indicated higher accuracy in localization compared to categorization. The stimulus effect [Δ*P*_*c*_ = 0.018, *F*_(1, 24)_ = 9.23, *p* < 0.01] indicated higher accuracy for houses compared to faces. There were no significant interactions.

### 3.3. Discussion of Experiment I

The b-CFS duration data showed that categorization took longer than localization. This result was consistently obtained for both object categories. Further, the data replicate a strong inversion effect for faces (Jiang et al., 2007; Stein et al., 2011a), independent of the task, but absence of inversion effects for houses, as also found recently (Zhou et al., 2010). The accuracy data showed consistently higher accuracy in localization compared to categorization. Hence, the results show that categorization was less accurate *and* that it took longer CFS epochs to reach the accuracy levels achieved in localization. These results imply that the longer CFS durations measured for categorization were not a result of decisional bias, whereby the observers operate on same performance levels for localization and categorization, reflected by same speed × accuracy products, but optimize one measure at the costs of the other. Yet, our data show no sign of a speed-accuracy trade-off, whereby shorter durations were achieved at the costs of more errors. In contrast, categorization performance was worse than localization in both measures, speed and accuracy, which indicates that not the same performance levels were reached in both tasks.

The categorization judgments are, in principle, prone to response bias, because response category and object category are confounded in categorization. However, ANOVA of the accuracy data showed no Task × Stimulus interaction. Looking at the observed accuracy advantage of houses compared to faces in the categorization tasks showed a modest advantage of just Δ*P*_*c*_ = 0.028, which is less than the rate of one error (which amounts *q*_*e*_ = 0.042). This means that the accuracy trade-off among the two object categories, which could rest on a potential response preference for houses, was negligible.

Methodologically, our results for the log-transformed durations confirm recent claims that transformations reducing positive skewness, thus reducing the weight of positive extremes, might be beneficial for revealing the effects of the experimental manipulations in the b-CFS paradigm (Gayet and Stein, 2017). However, generally, the results achieved with the raw duration measure and the log-transformed durations agree fairly well, indicating that conclusions about the experimental effects do not hinge on whether raw durations or transformed data are used.

## 4. Experiment II: terminated CFS

In Experiment II the same apparatus and the same stimuli were used as in Experiment I. Stimulus presentation was terminated by the computer after predefined durations, and the subjects judged location and category of the test stimulus consecutively in a dual task. Five CFS durations were used, which were found in pilot experimentation prior to the main experiment. These durations (50, 150, 750, 1,200, and 2,000 ms) were selected to sample the course of accuracy with increasing duration from chance to saturating performance. The choice of durations resembled the values used by Stein et al. (2011a), while we added one duration and extended the range to comprise one longer and two shorter durations.

### 4.1. Methods and materials

#### 4.1.1. Procedure

As in Experiment I the participants were briefed about dichoptic viewing conditions, adjusted seat and stereoscope to be able to maintain a comfortable position, and calibrated the stereoscope to ensure congruency of left and right eye displays. The participants were informed that brief periods of presentation would appear, and that they should try to guess stimulus localization and its object category as good as possible. Half of the subjects were instructed to consecutively indicate stimulus location relative to fixation and then object category, while the remainder subjects gave the two judgments in the reversed order. Before the experiment was started the participants went through 16 randomly selected practice trials with different stimulus material. The subjects received trial-by trial feedback about correctness after both judgments by a sequence of two well-separated tone signals. The event timings for displaying stimuli and CFS masker were exactly as in Experiment I. For each trial one of the five CFS durations was chosen at random. A trial was terminated by presenting black-white random dot patterns to the left and right eye display fields. The experiment was subdivided into four blocks which contained 80 trials each, each block comprising the same number of replications for stimulus categories, orientations and durations. In between these experimental blocks there was a brief pause for resting the eyes and to avoid overall fatigue.

#### 4.1.2. Data analysis and outlier clearing

The proportion correct measure was analyzed without any data clearing procedures. For localization, proportion correct was calculated from both response categories, “left” and “right.” This means that the proportion correct rates for face and house localization were not prone to potential decisional preferences of either response category. In contrast to localization, the two object categories coincide with the response alternatives in categorization. Therefore, the proportion correct rates for each object category are affected by a potential response bias to either faces or houses. In contrast to Experiment I, in which just 1–2 errors occurred on the average, accuracy variation in the whole range from chance to near perfect performance can be expected when the CFS intervals vary from brief to long. When error rates are no longer negligible, the influence of response bias toward either object category may be severe. That is, there could be substantial accuracy deviations among both object categories, but these deviations might reflect that accuracy for one object category is achieved at the costs of lower accuracy in the other object category due to decisional preferences. We therefore decided to calculate the proportion correct rates for categorization from both response categories to obtain an accuracy measure which was unaffected by decisional response preferences. This means that we analyzed performance in terms of proportion correct for three stimulus-response categories: face localization, house localization, and object categorization. The data were analyzed with a 5 (Duration) × 3 (Stimulus-Response Category; face localization, house localization, object categorization) × 2 (Orientation; upright or inverted) repeated measurements ANOVA.

Besides analyzing for performance differences in terms of the proportion of correct judgments we explored the course of the proportion correct rates along the chosen CFS durations with psychometric curve analysis. The objective of doing so was to obtain estimates of the critical durations which correspond to a 75% correctness criterion in order to obtain a first orientation how far the three stimulus-response categories may be separated in terms of b-CFS duration. Note that, for a complete analysis in terms of psychometric curves, more durations spread over a wider range of durations particularly for the longer durations may be necessary, since, otherwise, only a raw assessment of the saturation behavior of the curves is possible. We used the general form of the psychometric curve as outlined by Wichmann and Hill (2001), *P*_*c*_(*x*; *a, b*, γ, λ) = γ + (1 − γ − λ)*F*(*x*; *a, b*), whereby *a, b* were shape and scale parameter of the distribution function *F*, γ a guessing parameter, describing the proportion of correct responses in the absence of the signal, and λ the lapse rate, describing the rate of missing the correct response with full signal strength. The γ parameter was set to a fixed value of 0.5, since location and category judgments were obtained in two alternative forced choice. The remainder parameters entered an unconstrained least squares criterion estimation procedure with the Levenberg-Marquardt algorithm (Press et al., 1992). The Mathematica 11.0 (Wolfram Research) implementation of this algorithm was used. As a convenient choice for the distribution function, we used a gamma function, because the epoch durations in binocular rivalry typically follow a gamma distribution (Logothetis et al., 1996). Thus, the psychometric function had the form

(1) $$\begin{array}{r} P_{c}\left(D;a,b,\lambda \right)=0.5+\left(1-0.5-\lambda \right)\int_{0}^{D}t^{a-1}e^{-\frac{t}{b}}dt. \end{array}$$

### 4.2. Results

The rm-ANOVA on the proportion correct data revealed highly significant main effects of Duration [*F*_(4, 96)_ = 59.6, *p* < 0.001] and Stimulus-Response Category [*F*_(2, 48)_ = 9.91, *p* < 0.001], but no significant effect of Orientation [*F*_(1, 24)_ = 1.94, *p* = 0.177]. Further, there was a significant Stimulus-Response Category × Orientation interaction [*F*_(2, 48)_ = 3.56, *p* < 0.04]. The remaining interactions, all involving Duration, did not reach significance or marginal significance.

Exploring the Stimulus-Response Category × Orientation interaction with pairwise comparisons revealed a significant effect of orientation only for face localization [Δ*P*_*c*_ = 0.055, *F*_(1, 24)_ = 7.27, *p* < 0.01], but not for house localization [Δ*P*_*c*_ = 0.016, *F*_(1, 24)_ = 0.47, *p* = 0.498], and also not for categorization [Δ*P*_*c*_ = 0.008, *F*_(1, 24)_ = 0.43, *p* = 0.517]. Testing for performance differences with the three different stimulus-response categories revealed unique results for localization compared to categorization (see Table 3). For both stimulus categories, and in both orientations, there was a significant advantage of localization over categorization. This effect was pronounced and unique for the upright orientation of faces, while, for inverted stimuli, the advantage of face localization over categorization was just marginally significant. For houses, there was a pronounced and significant advantage of localization compared to categorization, which was independent of orientation. Face localization was more accurate than house localization for the upright orientation, but about equally accurate than house localization for inverted presentation^2^.

**Table 3:** Results of pairwise comparisons of face localization, house localization and object categorization performance for upright and inverted stimulus orientation.

	**Upright**						**Inverted**					
	**House localization**			**Categorization**			**House localization**			**Categorization**		
	***ΔP*_*c*_**	**F**	***p***	***ΔP*_*c*_**	***F***	***p***	***ΔP*_*c*_**	***F***	***p***	***ΔP*_*c*_**	***F***	***p***
Face-localization	0.044	3.01	0.096	0.081	17.24	0.000	−0.027	2.16	0.155	0.035	3.58	0.071
House-localization				0.045	5.03	0.034				0.061	12.40	0.002

*The table shows the difference in proportion correct, ΔP_c_, F-value, and significance level, p. All F- tests had 1 denominator and 24 nominator degrees of freedom*.

**Figure 3:**
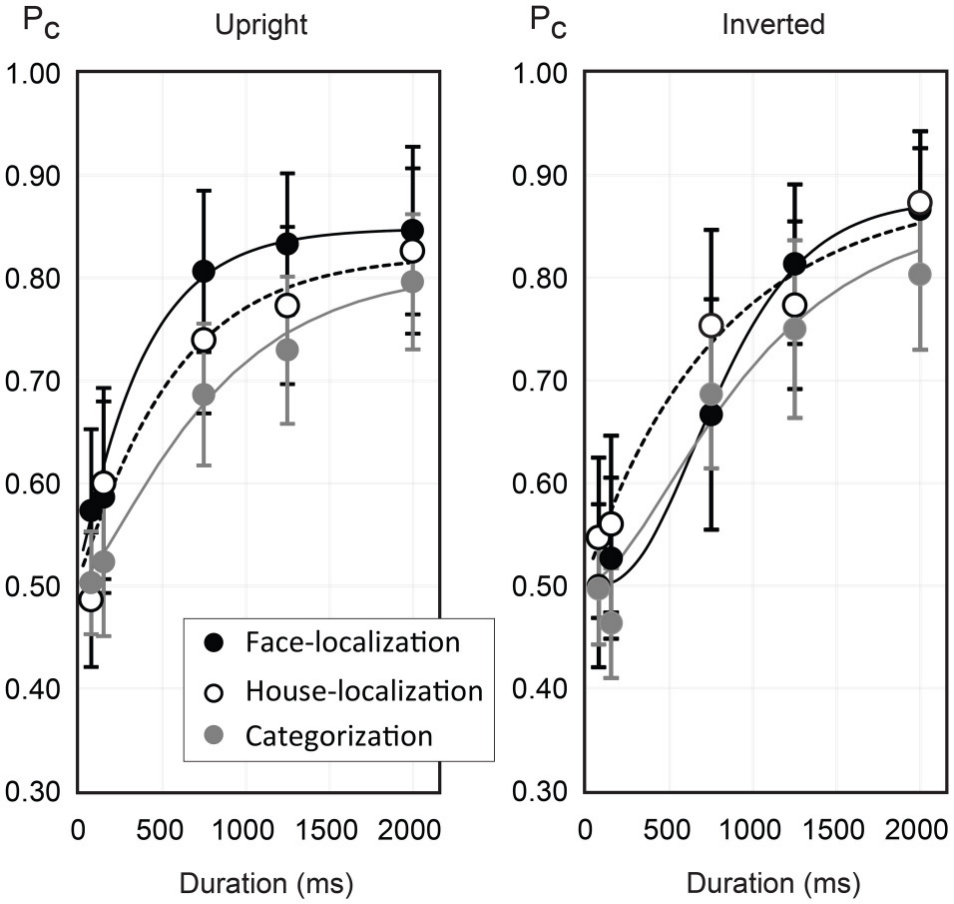
Results for Experiment II (terminated CFS). Mean portion correct rates are shown for localization of faces, localization of houses, and object categorization, for upright (left panel) and inverted presentation (right panel). The smooth lines indicate psychometric curves according to a gamma distribution function model. Error bars indicate the 95% confidence intervals of the means.

For all stimulus-response categories proportion correct rates rose monotonically with continuous flash duration, while the increase in performance slowed with increasing duration. For descriptive purposes we fitted the proportion correct data with psychometric curves of duration (see Methods for details of the chosen model and its parameters). The results of the parameter estimates are shown in Table 4, and the curves are shown in Figure 3 (smooth lines). Note that, for the gamma distribution, mean and variance are given by μ = *ab*, σ^2^ = *ab*^2^. Evaluation of goodness of fit showed that the ratio of explained to total variation, *R*^2^, was at least 0.95 (see 7th column of Table 4), which reflects high degrees of goodness of fit. However, the parameter estimates show clear deviations of the estimates for the lapse parameter obtained for upright and inverted presentation, which indicates different saturation behavior of the curve families for both orientations. While the curves for upright orientation show that leftward curve shift was associated with lower mean and standard deviation estimates, results for inverted presentation were not so unique^3^.

**Table 4:** Parameters of the psychometric curves for the gamma distribution function model (1), and extrapolated critical durations corresponding to a 75% correct criterion.

**Stimulus-response category**	**Orientation**	**λ**	***a***	***b***	***D*_0.75_**	***R*^2^**	**$\hat{\mu }$**	**$\hat{\sigma }$**
Face-localization	Upright	0.151	1.10	358.24	495.2	0.990	392.5	375.0
House-localization	Upright	0.176	1.14	498.63	836.3	0.953	568.8	532.5
categorization	Upright	0.191	1.54	534.91	1296.2	0.993	822.8	663.4
Face-localization	Inverted	0.124	3.86	226.05	990.9	0.995	872.8	444.2
House-localization	Inverted	0.112	0.94	871.20	834.9	0.978	816.3	843.3
categorization	Inverted	0.136	1.91	535.87	1216.9	0.964	1023.2	740.5

*The table shows lapse parameter, λ, shape parameter, a, scale parameter, b, critical 75% correct duration, D_0.75_, ratio of explained to total variation, R^2^, and estimated parameters for the gamma distribution, $\hat{\mu }$ and $\hat{\sigma }$*.

On the other hand, the psychometric curves fitted the proportion correct data smoothly in the range of [0.65,0.85], and were thus suited for estimating the critical durations, *D*_.75_, for which observers reached a 75% correct criterion (see 6th column of Table 4). The critical durations indicate that a 75% accuracy criterion in categorization was reached with substantial temporal delay compared to localization. This delay amounted about 460 ms compared to house localization and about 800 ms to face localization. This was true for the upright orientation. For inverted presentation, the difference in critical durations was not pronounced, but still amounted at least 200 ms.

### 4.3. Discussion of Experiment II

As in Experiment I, a clear advantage of localization over categorization was obtained in Experiment II. This was found for both faces and houses. Moreover, face localization, but not house localization was consistently better in the upright orientation, which entailed that the advantage of localization over categorization was larger and more unique in the upright orientation for faces, while it was about equal in both orientations for houses. This was found both in the accuracy measure, and also in the critical durations, *D*_0.75_, derived from the psychometric curves. Note that, in Experiment I, inversion effects for faces were observed in both tasks, while there were no inversion effects for houses in either task. Hence, when interpreting the results of Experiment II one should keep in mind that accuracy in categorization was calculated from the proportion correct rates of both object categories to avoid confounds with response bias. This, however, means that a potential face-specific inversion effect in categorization could not be revealed.

## 5. General discussion

In two experiments with the b-CFS paradigm we found that longer CFS durations were necessary to achieve the same accuracy in object categorization, compared to localization. These results imply that at the very moment the observer sees a stimulus breaking through CFS, she or he is still uncertain about its nature. The b-CFS duration estimates from Experiment I, in which accuracies indicated near perfect performance, and from Experiment II, in which critical durations corresponding to a proportion of 75% correct were estimated, together indicate that, roughly, basic level object categorization takes half a second longer to reach the performance level of localization under CFS conditions.

Our second major finding is a stable face inversion effect, while houses showed no inversion effects in both experiments. This replicates previous results obtained with the b-CFS method (see Introduction). Interpreting the inversion effect as a marker of higher level object related processing, the observation of a strong face inversion effect on the one hand and an advantage of localization over categorization on the other are somewhat paradox findings, since the latter finding might indicate that, at the edge of consciousness, lower level feature processing is more advanced than processing at the level of object category. We make a suggestion how this paradox may be resolved at the end of the discussion.

### 5.1. What does correct localization indicate?

There is a growing body of evidence proving brief periods of partial awareness in CFS. Recently, Gelbard-Sagiv et al. (2016) found evidence for a close link of lower level feature awareness and higher level unconscious processing. In a CFS priming paradigm there were priming effects for categorizing faces as famous or unknown only when subjects were able to indicate the color of the suppressed prime correctly, but not otherwise. The prime color was not a valid cue to face identity. This indicates that unconscious higher level face processing goes along with lower level feature awareness, while functional links among both do not necessarily exist. In former studies it was found that some basic stimulus attributes may escape suppression while others remain suppressed, indicating that not the unitary stimulus but rather dissociated aspects of the stimulus may enter awareness under CFS conditions. For example, subjects were able to locate the region where a flickering gabor was removed, but they could not indicate its orientation correctly (Zadbood et al., 2011). Similar findings were reported for the color of oriented bars, whereby color escaped the CFS masker, but bar orientation remained concealed (Hong and Blake, 2009). These findings cannot be explained by piecemeal rivalry with the CFS masker, since the escaping attributes remain “shapeless,” i.e., a locatable stimulus instance, or at least an excerpt of it, is not seen (Gelbard-Sagiv et al., 2016).

These findings reveal that the CFS method, initially designed to efficiently preclude probe stimuli from getting access to visual awareness, does not render complete suppression. This may be problematic, since in most studies only the attributes of interest are tested, while other, unnoticed stimulus properties with potential task relevance may escape from suppression. However, the lower level attributes, which were reported to do so, were no valid predictors of the higher level object attributes under scrutiny (Gelbard-Sagiv et al., 2016). The b-CFS technique tests the first moment in time at which the observer can locate the test stimulus. Well, the findings about lower-level feature escaping from suppression (s.a.) may cast into doubt that b-CFS durations indicate the first moment in time the observer sees “something other” than the flickering Mondrian. However, our finding that correct categorization is possible only at later moments after localization implies that no higher level cues about object identity are conveyed in potential leaks of CFS. Further, the temporal delay of categorization relative to localization suggests smooth transitions in the first break of CFS, in which the observer first sees localizable fragments of the stimulus emerging in the Mondrian, and then, after more fragments are added or the fragments enlarge, she/ he is able to judge its nature. The results of both experiments indicate that about half a second after valid spatial cues to object presence emerge there is enough evidence to judge the objects' category. This suggests that the breaking CFS event proceeds unidirectional in time. The two judgments occur at delayed moments, after the observer's evidence collection about the stimulus has reached clearly different states. The tight temporal coupling of localization and categorization found in both experiments is evidence that the ability to localize the stimulus correctly in b-CFS is not due to a transient temporal leak of CFS, where dissociated low-level stimulus attributes shine through the masker. Instead, this shows that the moment in time at which the observer localizes the test stimulus correctly marks the start of its non-reversible transition into awareness.

### 5.2. Knowing “where” and knowing “what”

Studying object recognition under under normal binocular viewing conditions has revealed conflicting findings about the question whether object detection precedes categorization. Using a backward masking paradigm with briefly flashed objects of variable durations, human observers were at the same degree of accuracy for judging whether an image contained an object or not, and for indicating its basic category (e.g., face, house, car, tree). For correct within-category discrimination/identification (e.g., Sean Connery, bungalow, porsche, oak) longer durations were necessary (Grill-Spector and Kanwisher, 2005). These findings suggested that figure-ground segmentation and object categorization are handled at the same level of processing in the hierarchy. Support for this claim came from related findings on ultra-rapid object detection in visual scenes, which suggest that category-specific signatures in neural recordings can be derived from the initial wave of activation, as early as 120–130 ms after stimulus onset (Kirchner and Thorpe, 2006; Thorpe, 2009).

While the coincidence of detection and categorization holds for whole and intact objects, categorization proved worse than object detection when degraded objects were used (Mack et al., 2008). Similarly, experiments on texture figure perception in noisy surrounds uniquely showed that detection of object presence is possible with relatively low feature contrasts of figure and surround, while larger feature contrasts are necessary to reach the same levels of accuracy in shape discrimination tasks (Meinhardt et al., 2006; Persike and Meinhardt, 2008)^4^. Thus, the finding of a temporal delay of object categorization compared to localization in b-CFS indicates that the transition of the test stimulus into conscious perception somehow resembles object detection and shape discrimination in noise, but not object vision in unmasked natural scenes. Apparently, knowing “where” does not imply knowing “what” under CFS conditions. That is, there is no awareness of the basic level object category in the moment the observer notices stimulus presence.

### 5.3. The specificity of findings under CFS conditions

In the seminal study about the face inversion effect in b-CFS (Jiang et al., 2007) the authors included a binocular control condition, where the CFS masker was presented to both eyes and the test faces were faded into the masks, increasing contrast linearly with time. This “no-suppression” condition was included to prove whether higher level object processing is specific to unconscious processing under CFS conditions. This control technique was applied in several consecutive studies (Costello et al., 2009; Zhou et al., 2010; Mudrik et al., 2011; Stein et al., 2011a; Stein and Sterzer, 2012).

Jiang et al. (2007) found face inversion effects in b-CFS, but not in the binocular control condition. This led to the conclusion that the inversion effect is a unique marker of unconscious high-level processing under suppression conditions. The finding was challenging to the prevailing notion that suppression blocks information before reaching the late, object tuned stages in the ventral stream (Tong et al., 1998; Tong and Engel, 2001). In a series of experiments Stein et al. (2011a) employed several varieties of the binocular control condition, and obtained the face inversion effect both in b-CFS and in most varieties of the control condition. This led to a debate about the use of the binocular control condition [see comprehensive discussion in Yang et al. (2014) and Stein et al. (2011a)], in which Yang et al. (2014) made an important conceptual point. For each finding obtained in b-CFS one may ask why this finding should be unique to unconscious processing under CFS conditions. The face inversion effect, which is obtained in conscious processing with several experimental paradigms (see Introduction), may also be obtained in the binocular control condition, as shown by Stein et al. (2011a). This finding is plausible, given findings that upright faces are efficiently found in complex visual scenes (Hershler and Hochstein, 2005), and higher-level attributes, such as face familiarity, add a search advantage (Persike et al., 2013). Since the control condition sets up conditions likewise detection of objects in noise, an advantage of upright faces is again plausible given findings that segmentation can be guided by object knowledge (Peterson and Gibson, 1993, 1994; Peterson and Kim, 2001). It is important to note that whether the inversion effect is found or not in the control condition tells us something about processing in the control condition, but hardly anything about processing in the CFS condition. Running a binocular control condition cannot help us much in deciding whether there is unconscious higher level object processing under suppression conditions. Much more crucial for this claim is the question which kind of features may escape from suppression in potential leaks of CFS. If low or mid-level features that are linked to the indicators used to infer higher level unconscious processing escape from suppression, this would cast doubts that different b-CFS durations truly reflect differences in unconscious processing of the high-level attributes under scrutiny. This, however, remains to be shown (see Section 5.1).

### 5.4. Implications for the site of competition

We stated at the beginning of the Discussion that the face inversion effect on the one hand and the advantage of localization over categorization on the other is a somewhat paradox finding. However, unconscious processing of the object under suppression does not imply that categorial information is made available in the moment when CFS is broken. In one prominent view of binocular rivalry, local, low-level competition in early retinotopic feature-selective areas is essential for binocular rivalry (Tong et al., 2006). The local-low level competition, however, is conceived to be modulated by top-down influence from higher visual areas of the ventral stream. The neural basis of unconscious face-tuned processing remains somewhat obscure, since there is evidence that activity in ventral face-selective regions is weak in the suppression period of rivalry (Tong et al., 1998; Tong and Engel, 2001). A recently discovered subcortical route that starts in the superior colliculus and projects to the amygdala may be partly involved in rivalry of faces and objects (Pasley et al., 2004), but it is not likely to underly the processing advantage of upright faces due to its relatively primitive pattern vision mechanism (Pessoa et al., 2002a,b). Albeit weak activity, Jiang and He (2006) showed that neural activity in high level areas specific for face processing can distinguish faces from scrambled versions of the latter under conditions of interocular suppression. Using CFS, Sterzer et al. (2008) were able to show that object category was predictable by multivariate pattern analysis from activity in brain regions responsible for higher level visual processing, albeit with 58–63% accuracy during 600 ms of presentation of low contrast faces and houses, yet significant above chance. These results suggest that the activity in face and object-tuned areas of the ventral stream arising in the suppression epoch of rivalry may be sufficient to trigger earlier dominance change in the competition of the local, low level feature tuned mechanisms in earlier layers. If the transition is mediated by local retinotopic mechanisms, a nature of transition with a “patchy” appearance of the test stimulus in the masker, where the patches grow in time and become more frequent, is plausible (see, e.g., Figure 1 in Tsuchiya et al., 2006). This process may start earlier if top-down modulation from higher level areas biases competition toward known objects or upright faces. Moreover, if the transition proceeds on the level of the early, local mechanisms, segmentation precedes integration into shapes and objects in a similar way as it is observed for the detection and discrimination of shapes in cluttered images. As a result, categorization should become possible after the observer sees “something” breaking through CFS. Conscious access to the cause of the early trigger of the transition process does not need to be involved.

## Author contributions

All authors contributed equally to conception and design of the study. MP programmed all experminetal routines. FK conducted the experiments. GM and FK contributed data analysis and interpretation. All authors were involved in writing, preparation of the manuscript and its final approval. All authors agree to be accountable for all aspects of the work in ensuring that questions related to the accuracy or integrity of any part of the work are appropriately investigated and resolved.

### Conflict of interest statement

The authors declare that the research was conducted in the absence of any commercial or financial relationships that could be construed as a potential conflict of interest.

## Footnotes

^1^The authors used a priming paradigm to measure the effect of face familiarity in CFS. The study was reported on VSS 2013, and is, to our knowledge, the first study reporting that judgments of stimulus localization and judgments of object-related properties require different processing times under CFS conditions.

^2^Correcting the α level for the effects of multiple independent comparisons (Bonferroni corrected alpha level) yields a test-alpha of $\hat{\alpha }=1-{\left(1-\alpha \right)}^{1/m}\approx \alpha /m$, whereby *m* is the number of comparisons (c. Johnson and Wichern, 2002, p. 232). Applying this correction for an assumed overall alpha level of α = 0.05 for *m* = 4 tests leads to $\hat{\alpha }=0.0125$, which means that the advantage of house localization over categorization cannot be regarded as significant any more.

^3^As outlined by Wichmann and Hill (2001), the correct estimate of the lapse parameter is crucial for the correct estimates of all other parameters of the psychometric function. The divergent estimates of the lapse parameter for upright and inverted presentation might potentially vanish if more data for longer presentation times were available. Estimation of psychometric curves for b-CFS data has so far not been addressed, and would require a study explicitly dedicated to this issue, since particularly the longer durations and its variability across subjects is an intricate problem (see also Gayet and Stein, 2017). Note that the major purpose of our psychometric curve estimation was to obtain a first assessment of the durations corresponding to a 75% correctness criterion.

^4^In the shape discrimination tasks used by Meinhardt and colleagues the observers judged elementary shape differences, i.e., whether a lozenge was leftward or rightward tilted, or whether the figure had just rectangular or also oblique elements. For these judgments more feature contrast was necessary compared to indicating whether there was a texture figure hidden in the cluttered surround (detection). At the feature contrasts where observers could well indicate that a texture figure was present, they could only poorly judge about its shape (see Figure 5 in Meinhardt et al., 2006 and Figures 4, 5 in Persike and Meinhardt, 2008).
